# Body exposure and vocal analysis: validation of fundamental frequency as a correlate of emotional arousal and valence

**DOI:** 10.3389/fpsyt.2023.1087548

**Published:** 2023-05-24

**Authors:** Vanessa Opladen, Julia A. Tanck, Julia Baur, Andrea S. Hartmann, Jennifer Svaldi, Silja Vocks

**Affiliations:** ^1^Department of Clinical Psychology and Psychotherapy, Osnabrück University, Osnabrück, Germany; ^2^Department of Clinical Psychology and Psychotherapy, University of Tübingen, Tübingen, Germany; ^3^Department of Psychology, Experimental Clinical Psychology, University of Konstanz, Konstanz, Germany

**Keywords:** fundamental frequency, paralinguistic, psychophysiology, body exposure, body image

## Abstract

**Introduction:**

Vocal analysis of fundamental frequency (*f*0) represents a suitable index to assess emotional activation. However, although *f*0 has often been used as an indicator of emotional arousal and different affective states, its psychometric properties are unclear. Specifically, there is uncertainty regarding the validity of the indices of *f*0_*mean*_ and *f*0_*variabilitymeasures*_ (*f*0_*dispersion*_, *f*0_*range*_, and *f*0_*SD*_) and whether higher or lower *f*0 indices are associated with higher arousal in stressful situations. The present study therefore aimed to validate *f*0 as a marker of vocally encoded emotional arousal, valence, and body-related distress during body exposure as a psychological stressor.

**Methods:**

*N* = 73 female participants first underwent a 3-min, non-activating neutral reference condition, followed by a 7-min activating body exposure condition. Participants completed questionnaires on affect (i.e., arousal, valence, body-related distress), and their voice data and heart rate (HR) were recorded continuously. Vocal analyses were performed using Praat, a program for extracting paralinguistic measures from spoken audio.

**Results:**

The results revealed no effects for *f*0 and state body dissatisfaction or general affect. *F*0_*mean*_ correlated positively with self-reported arousal and negatively with valence, but was not correlated with HR_*mean/maximum*_. No correlations with any measure were found for any *f*0_*variabililtymeasures*_.

**Discussion:**

Given the promising findings regarding *f*0_*mean*_ for arousal and valence and the inconclusive findings regarding *f*0 as a marker of general affect and body-related distress, it may be assumed that *f*0_*mean*_ represents a valid global marker of emotional arousal and valence rather than of concrete body-related distress. In view of the present findings regarding the validity of *f*0, it may be suggested that *f*0_*mean*_, but not *f*0_*variabilitymeasures*_, can be used to assess emotional arousal and valence in addition to self-report measures, which is less intrusive than conventional psychophysiological measures.

## Introduction

Verbally expressing one’s emotions and understanding the affective responses of others are central to human communication. To assess the expression of affect, many studies [e.g., (1–3)] have integrated a two-dimensional approach splitting affect into arousal [level of physiological awareness; (4)] and valence [level of pleasure/displeasure; (5)]. While there are several well-validated questionnaires to measure both arousal and valence, the reliance on questionnaire data can entail a risk of self-report bias (6, 7). Further, a lack of emotional introspection or interoception in the participant may bias the data [e.g., (8)]. A more objective bodily indicator to measure affect is the use of psychophysiological indices [e.g., blood biomarkers, heart rate (HR), electrodermal activity or endocrine parameters]. Such markers are often applied in research in order to generate more objective data (9). However, although these psychophysiological measures are less subjective than self-report questionnaire measures, they likewise appear to come with a risk of bias: Due to their salience and visibility, they are likely to distract participants from the task at hand (6). Moreover, the invasive nature of some methods, such as the collection of blood markers, decreases participants’ compliance (10) and physical comfort (11). In addition, some psychophysiological measures are likely to cause artifacts due to the draping of wires and the restriction of participants’ mobility [cf. (12)]. These limitations of psychophysiological methods likely contribute to the low correspondence of psychophysiological measures among each other (13) and with subjective data (14).

Vocal analysis, as a well-established tool in clinical psychology (15), may counteract some of the disadvantages of psychophysiological measures. A particular quality of vocal analysis is that voice data can be derived from audio recordings (16), rendering the method user-friendly for the participant. Moreover, given its non-invasive nature (17), vocal analysis may potentially reduce the bias that is inherent in measuring affect using other psychophysiological methods (16). Fundamental frequency (*f*0) is a commonly used instrument to examine affect by means of the voice (18). *F*0 is the measurable substrate with which the perceived vocal pitch is highly correlated, and refers to the vibration of vocal folds (19). It physically represents the lowest vocal frequency harmonic of a waveform measured in Hertz (Hz); (20). Under the assumption that *f*0 is an indicator of vocally encoded emotional arousal [e.g., (21, 22)], it has been examined in a variety of different contexts. For example, *f*0 has been viewed as an indicator of arousal in the context of discussions in romantic relationships [(23); i.e., *f*0_*mean*_] or family conflicts [(24); i.e., *f*0_*range*_] and has also been investigated as an indicator of stress [(25); i.e., *f*0_*range*_], empathy [(26); i.e., *f*0_*mean*_] or to detect clinical social anxiety [(27); i.e., *f*0_*mean*_]. Besides this, numerous studies have suggested that *f*0 might represent a marker of specific emotional states (28) such as fear [e.g., (29); i.e., *f*0_*mean;*_ (30); i.e., *f*0_*range*_]. Other studies found no difference in *f*0 between diametrically opposed emotions such as happiness and fear [(31); i.e., *f*0_*mean*_ and adapted *f*0_*range*_], thus calling into question the suitability of deriving different emotions from *f*0. If not as a marker of a single emotion, but as a marker of the dimension of valence [pleasant, unpleasant; (5)], *f*0 has received less research attention (32) and there is little (if any) agreement on whether *f*0 is associated with valence in general [cf. (33)]. Therefore, while *f*0 has been studied in many contexts, it has not been directly validated as a marker of arousal. Likewise, while it has been examined with regard to specific emotional states, it has not yet been directly validated as a broad marker of valence.

Furthermore, the question of which distributional characteristic of *f*0 fits to examine affect remains unanswered. Two debated parameters described in literature are *f*0_mean_ [e.g., (34, 35)] and *f*0_variabilitymeasures_ [e.g., (30)]. *F*0_mean_ refers to the arithmetic mean of *f*0. As the most common statistical measure used to indicate the central tendency of a distribution (36), it refers in this case to the interval-scaled variable of *f*0, and it is calculated as the sum of all measured values divided by the number of values (36). Regarding *f*0_variabilitymeasures_, we refer to the statistical indices of *f*0_range_ (i.e., *f*0_max_–*f*0_min_) and *f*0_SD._ By using the term *f*0_dispersion,_ we refer to an adapted range, because the usual calculation of *f*0_range_ might bias information about the *f*0 distribution in the case of natural outliers (29). Therefore, as described in Hirst (37), *f*0_dispersion_ displays the calculated difference between the largest and the smallest measured value with a cut of the 0.1 and the 0.2 quantile from the top and bottom *f*0. It is debatable whether *f*0_mean_ (34, 35) or *f*0_variabilitymeasures_ (30) are more valid to detect arousal and valence in acoustic features. Both indices seem reasonable, as they have been generally found to be markers of affect [for an overview see (28)]. However, in terms of direction, arousal and valence have been reported to be related to both higher *f*0_mean_ (19, 38) and higher *f*0_variabilitymeasures_ (39), as well as lower *f*0_mean_ (34) and lower *f*0_variabilitymeasures_ (30, 40). For instance, Rothkrantz and colleagues (38) designed an experiment in which cognitive workload was induced using different stress provoking tasks (e.g., Stroop test) and found an increase in *f*0_mean_ and *f*0_variability_ with heightened levels of emotional stress. Likewise, Lively and colleagues (40) induced emotional stress in their participants using a visual tracking task to manipulate cognitive workload. However, in this experiment, the authors found a decrease in *f*0_variability_ and no consistent effect for *f*0_mean_. Therefore, although both tasks were equally stress-provoking, the outcome regarding *f*0 was ambivalent. Thus, it is unclear whether affect is associated with higher (19, 24, 41, 42) or lower (30, 43, 44) *f*0_mean_ and *f*0_variabilitymeasures_.

To sum up, *f*0 has not yet been directly validated as a correlate of affect (i.e., arousal and valence). Moreover, its underlying dimensions of arousal and valence as well as the significance of high and low *f*0 indices are yet to be examined. The domain of body image might be a suitable research field to resolve this uncertainty and to further validate *f*0, as real-time measurements are of importance in this field: On the one hand, given that body image is known to have a trait-like and a state component (45), prospectively or retrospectively assessed questionnaire data might be biased due to natural state fluctuations in body image (46). On the other hand, non-invasive psychophysiological measurements may be useful in the field of body image. As the main stimulus or stressor is often the subject’s own body [e.g., (47)], visible psychophysiological measures applied on the body (e.g., electroencephalogram) may be distracting and might directly influence the validity of the respective studies. In the clinical context, body exposure is a commonly used technique to improve body image, in which individuals are instructed to look at their body while verbalizing the arising thoughts and emotions (48). Body exposure is therefore suitable to create physiological affective reactions [cf. (49)], as it has been shown to create arousal (50–52) and body-related distress (49) according to self-reported questionnaire data, including in healthy populations (47, 50).

Underlining the importance of non-invasive measures in the field of body image, two studies have already examined the predictive value of *f*0 as a correlate of body-related distress during a body exposure task (34, 35). However, in line with the aforementioned ambiguity of previous research, the results differed according to the respective sample of each study: *F*0_mean_ was found to be positively related to the construct of state body dissatisfaction in a sample of female participants with overweight and obesity (35) but unrelated to the same construct in a sample of women with binge eating disorder (34). The authors explained this discrepancy by a lack of ability of individuals with eating pathology to adequately engage physiologically in tasks that provoke body-related distress (34). In both studies, *f*0 was assessed only as a correlate of body dissatisfaction and not as a correlate of affect or its underlying dimensions arousal and valence (3, 33). Besides the fact that the above-mentioned studies exclusively focused on body-related distress, they also lacked detailed analyses of other metrics: Contrary to recommendations [cf. (53)], additional vocal indices (e.g., *f*0_varability measures_) and the connection to different physiological measures such as HR (34) have not yet been discussed in the context of body exposure. As such, indications that *f*0 represents a marker of vocally encoded affect, arousal, valence, and potentially body-related distress, remain scarce.

In the present study, we therefore aimed to validate the indices of *f*0_mean_ and *f*0_varability measures_ (*f*0_dispersion_, *f*0_range,_ *f*0_SD_) as correlates of vocally encoded emotional arousal, valence, and body-related distress (i.e., trait-like eating disorder severity and state body dissatisfaction) during body exposure in healthy women. To examine psychophysiological activation (i.e., valence, arousal, body-related distress), we used voice and HR data from a 7-min body exposure session in which participants looked at their body and freely described their body-related thoughts and feelings. We compared this body exposure (experimental) condition to a preceding neutral, non-body-related baseline (control) condition. Trait-like eating disorder severity was assessed directly before participants underwent the stressor of body exposure. As state measures, we administered self-report questionnaires on state body dissatisfaction, arousal, valence, and general affect before, (during), and after the body exposure.

Despite the ambiguity regarding the direction of *f*0, in our first hypothesis, we expected an increase in *f*0_mean_ during the body exposure condition compared to the baseline condition, in line with previous studies on vocally encoded body-related distress (34, 35). Moreover, based on studies in patients with anxiety disorder [e.g., (30)], we expected a decrease in *f*0_variabilitymeasures_ during the body exposure condition compared to the baseline condition. Second, in accordance with findings by Baur and colleagues (35), we hypothesized that body exposure would induce more arousal for individuals with higher trait-like eating disorder severity, which should be reflected in increased *f*0_mean_ and decreased *f*0_variabilitymeasures_. Third, in line with the positive correlations between *f*0 and questionnaire-based pathology reported in patients with anxiety disorder (54), for state measures, we hypothesized positive correlations of *f*0_mean/variability measures_ with state body-related distress, self-reported arousal, and negative correlations with self-reported valence and general negative affect. Fourth, also in terms of convergent validity, we hypothesized significant positive correlations between *f*0_mean/variability measures_ and the psychophysiological marker of HR_mean/maximum_. Further, in terms of comparability between the two psychophysiological measures, we assumed that the HR would follow the expected pattern of an increase during body exposure, as also hypothesized for *f*0_mean/variability measures_.

## Materials and methods

### Participants

The study was approved by the local Ethics Committee (4/71043.5). The sample was community-based and recruited by means of the local university’s mailing list, social media advertisements, as well as personal contacts. The inclusion criteria were identifying as female and an age between 18 and 45 years, and the exclusion criteria were self-reported current or past diagnosis of a mental disorder, history of and current drug abuse or acute intoxication by psychotropic substances, and past or present suicidal tendencies or self-harm behavior. We only included participants who identified as female, as this population is likely to show greater body dissatisfaction than, for example, participants who identify as men (55), and we therefore expected higher stress responsiveness in females than in a mixed-gender sample. Moreover, due to potential natural variations in *f*0 between different genders [i.e., higher in females; (42) and lower in males, (56)], it was important for the comparability of the data to remain within the range of a female *f*0. Recruitment began with a first email contact and prospective participants subsequently underwent a structured telephone screening to check the inclusion and exclusion criteria. Out of 113 initial email contacts, *n* = 2 participants did not meet the inclusion criteria, *n* = 21 reported no further interest in participating, and *n* = 13 did not respond to any contact attempts. During the course of the study, *n* = 2 participants dropped out and *n* = 1 declared a diagnosed eating disorder in remission after testing. During the analysis, *n* = 1 participant was excluded due to missing data. Therefore, data from *N* = 73 female participants were ultimately analyzed. As reimbursement, participants received course credits or a €5 gift voucher per hour of participation.

### Psychological measures

#### Trait-like measures

##### Sociodemographic and study-relevant characteristics

Participants provided information on basic sociodemographic data such as age, nationality, employment status, education, and body-related personal data such as hours of exercise including weight-training weight-training, dieting, and therapeutic treatment. The body mass index (BMI) was retrospectively calculated by dividing self-reported weight (in kg) by height squared (in m^2^).

##### Eating Disorder Examination-Questionnaire

The Eating Disorder Examination-Questionnaire [(EDE-Q); (57, 58)] is a trait-like instrument assessing the frequency and severity of eating disorder symptoms. It comprises 22 items divided across four subscales: Restraint, Eating Concern, Weight Concern, Shape Concern. Items are rated on a seven-point Likert scale (from 0 = *no days/none of the time/not at all* to 6 = *every day/every time/markedly*). Internal consistencies were found to be good to excellent in a validation study [α = 0.97 for the global score; 0.85 < α < 0.93 for the separate subscales; (59)] and in the present study (α = 0.92 for the global score, 0.76 < α < 0.86 for the separate subscales).

##### Eating Disorder Inventory-2

The Eating Disorder Inventory-2 [(EDI-2); (60, 61)] is a self-report instrument measuring trait-like eating disorder severity. In the present study, we used the two subscales Body Dissatisfaction (nine items) and Drive for Thinness (seven items) to assess the participants’ (dis)satisfaction with body parts and preoccupation with their body. All items are rated on a 6-point Likert scale (from 1 = *never* to 6 = *always*). Previous studies in healthy females have demonstrated excellent Cronbach’s α for both subscales [Body Dissatisfaction: α = 0.90, (62); Drive for Thinness: α > 0.86, (63)], as did the present study (Body Dissatisfaction α = 0.84; Drive for Thinness Scale α = 0.88)].

#### State measures

##### Body Image States Scale

The Body Image States Scale [(BISS); (45, 64)] was used to assess cognitive-affective changes in state body dissatisfaction. The BISS contains six items assessing current (dis)satisfaction with one’s physical appearance on a nine-point Likert scale (from 1 = *extremely dissatisfied* to 9 = *extremely satisfied*). In a previous study in healthy females (55), internal consistency ranged from good to excellent (0.82 < α < 0.90), which was also the case in the present study (0.89 < α < 0.91).

##### Self-Assessment Manikin

The Self-Assessment Manikin [(SAM); (65)] is a picture-based instrument in which participants rate the broad dimensions of Arousal and Valence on the depicted figures. In the present study, the SAM was used as a state instrument (i.e., directly before, during, and directly after body exposure). Participants performed single ratings on a nine-point Likert scale (from 1 = *extremely calm* to 9 = *extremely aroused* for Arousal and from 1 = *extremely unpleasant* to 9 = *extremely pleasant* for Valence). In a previous study in a population of individuals without mental disorders, Cronbach’s α values were excellent to acceptable [α = 0.98 for Arousal, and α = 0.63 for Valence; (66)]. In the present study, both dimensions showed excellent Cronbach’s α values (α = 0.90 for Arousal and α = 0.89 for Valence).

##### Positive and Negative Affect Schedule–Expanded Form

To assess self-reported general affect in relation to one’s body, the Positive and Negative Affect Schedule–Expanded Form [(PANAS-X); (67, 68)] was applied as a state measure. The General Negative Affect scale and the General Positive Affect scale each contain 10 items rated on a five-point Likert scale (1 = *not at all* to 5 = *extremely*). The German version of the PANAS-X has proven to be highly internal consistent for both subscales [0.77 < α < 0.92; (68)]. Internal consistency in the present study was in a similar range (0.69 < α < 0.78 for General Negative Affect; 0.86 < α < 0.90 for General Positive Affect).

#### Physiological measures

##### Fundamental frequency: vocally encoded emotional arousal and valence

**FIGURE 1:**
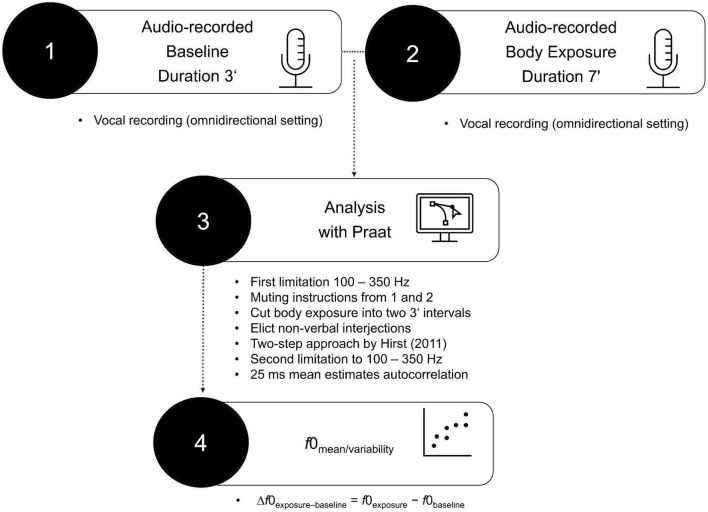
Procedure of vocal analysis.

*F*0 (in Hz) in the baseline condition and the body exposure condition was analyzed using Praat, a free-of-charge speech analysis program (69). The procedure of vocal analysis is depicted in Figure 1. Before examining *f*0, default settings limited the *f*0 range from 100 to 350 Hz, which corresponds to the usual female speaking voice (70). Next, the previously recorded instructions (lasting for 1 min) were muted on the tape in both conditions, leaving pure participant voice data for the baseline condition (3 min) and for the body exposure condition (6 min). Using the free audio editor Audacity 2.1.2 (71), the remaining voice data in the body exposure condition were cut into two 3-min intervals in order to facilitate the comparability with the baseline data within subsequent autocorrelation estimates. In a next step, using Praat, we manually eliminated non-verbal interjections (i.e., coughing, exhaling, throat-clearing), ambient noise (i.e., mouse clicking), and periodicity (i.e., existing algorithm without corresponding voice) to improve data quality. The specific excluded noises are shown in Table 1. A further *f*0 adaptation was implemented using the two-step approach suggested by Hirst (37). To further ensure that an individual’s range still corresponded to the usual female vocal range of 100 to 350 Hz, following the procedure of Hirst (37), an additional top and bottom limitation was added.

**TABLE 1:** Specific eliminated noises of voice recordings.

Voice		Noise		
**Participant**	**Human**	**Environmental**	**Technical**	**Artifact**
Coughing ^(3)^	Technician ^(2)^	Mouse click ^(7)^	PC sound ^(2)^	Hum ^(21)^
Vocal sound ^(5)^	Laughing ^(2)^	Rustle ^(12)^	Microphone ^(1)^	Creak ^(2)^
Laughing ^(20)^	Coughing ^(2)^	Melody ^(2)^	Telephone ^(1)^	
Throat-clearing ^(5)^	Throat-clearing ^(3)^	Bell ^(1)^		
Breathing ^(7)^	Breathing ^(2)^	Dull sound ^(3)^		
Interjection ^(7)^		Chairs ^(1)^		
Yawning ^(13)^				
Nose-blowing ^(5)^				
Inhale ^(10)^				
Exhale ^(10)^				
Question ^(1)^				
Smacking one’s lips ^(2)^				

Numbers in brackets depict the number of events (*N* = 73).

For all audio data, mean *f*0 estimates for each 25 ms were established using autocorrelation methods provided in Praat, resulting in an *f*0 score for each participant for the baseline and the body exposure condition. Following Baur and colleagues (35), it was necessary to calculate the *f*0 baseline for each person separately in order to control for pre-existing individual vocal differences. As a type of baseline centering (16), the calculated difference scores were assumed to depict the participants’ change in vocally encoded emotional arousal from baseline to body exposure (i.e., Δ*f*0_exposure–baseline_ = *f*0_exposure_–*f*0_baseline_).

##### Heart rate

Heart rate (in beats per minute; bpm) was assessed using an HR monitor (i.e., Garmin Vivosmart 4) worn on the participants’ left wrist. Participants told the instructor the time they started and ended each condition, such that a trigger was set and the HR monitor was paused when a new state measure was to be completed. Analogous to *f*0 [cf. (35)] and to account for individual differences in HR, mean difference scores in the body exposure condition relative to baseline were calculated (i.e., ΔHR_exposure–baseline_ = HR_exposure_–HR_baseline_). To draw from different indices, HR was assessed using two commonly used parameters, that is HR_mean_ (72) and HR_maximum_ (73). HR_mean_ describes the arithmetic mean of the HR interval while HR_maximum_ depicts the highest HR value of the HR interval.

#### Experimental conditions

**FIGURE 2:**
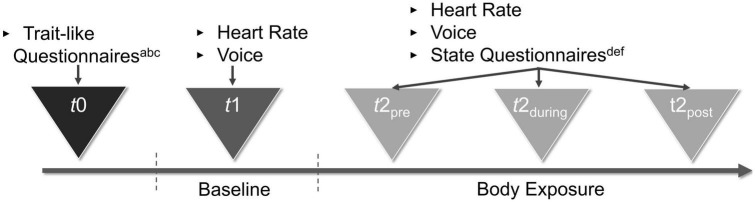
Procedure of the study. ^a^Sociodemographic Characteristics. ^b^Eating Disorder Examination-Questionnaire. ^c^Eating Disorder Inventory–2. ^d^Body Image States Scale. ^e^Self-Assessment Manikin. ^f^Positive and Negative Affect Schedule–Expanded Form.

The procedure of the present study was structured into a two-part repeated measures design consisting of baseline and a body exposure session (as depicted in Figure 2). The 3-min baseline measure served the purpose of using voice and HR as a reference for the body exposure condition. During the baseline measure, participants were asked to describe out loud nine neutrally validated pictures from the Open Affective Standard Image Set [(OASIS); (74)] database, which were hung at the top of a curtain in the mirror cabin. The instructions for the baseline condition were as follows: “For the next 3 min, please describe the nine pictures you see right in front of you. The accuracy of your statements is not important; all that matters is the recording of your voice and HR. It does not matter which images you describe in which order. We ask that you speak for the entire time. You are welcome to repeat sentences […].” During the baseline condition, participants wore their everyday clothes, and the mirror sides of the cabin were covered with a white curtain to avoid distraction. Subsequently, the experimental condition of body exposure with non-guided verbalization [cf. (47)] was implemented. During 7-min sessions encompassing 1 min of standardized audio instructions and 6 min of verbalization, participants were asked to freely reflect on their body-related cognitions and affect. The instructions for the experimental condition were as follows: “This exercise is about freely talking out loud about your thoughts and feelings about your body. There is no right or wrong way to do this; it is all about your feelings and thoughts. You will hear a tone signal right away, after which you should begin to talk about your thoughts […].” In the experimental condition, participants undressed to their underwear and the curtain of the mirror cabin was removed such that participants viewed their entire body from the front, back, and both side angles.

### Mirror cabin equipment

The standardized settings consisted of a three-sided mirror cabin designed for the purpose of the study, including a microphone (i.e., type: t.bone SC1100, Thomann GmbH Germany; setting: omnidirectional) and an LED light inside. Both the baseline and the body exposure condition took place in this cabin, which had a height of 2.12 m and a width of 0.85 m for each of the three sides, enabling the participants to look at themselves from all angles.

### Procedure

The study was part of a larger experiment whose procedure is described elsewhere (47) and consisted of an additional three 48-min body exposure sessions that followed the present experiment. Data were gathered in two identically equipped laboratories of the local university. The general setup is depicted in Figure 2 and was as follows: First, the rationale of exposure was described, and participants were informed about the goals of the study and provided informed consent. Next, the participants completed the trait-like and state questionnaires. Subsequently, the HR monitor was placed around their wrist and they entered the mirror cabin. To protect their privacy, participants were alone in the cabin while a graduate student provided assistance, if necessary, from behind a screen. Then, the microphone and the HR monitor were started for continuous recording and the baseline condition was completed. First the baseline and then the body exposure condition took place. After completing the full experiment, participants got dressed and were shortly debriefed. In addition, they were able to talk about their mental state. For the purpose of standardization, all instructions in the baseline and body exposure conditions were played as pre-recorded audio instructions. When completing the paper-and-pencil measures on general state affect and state body-related distress before and after the baseline and the body exposure condition, the participants wore a bathrobe. State arousal and valence were assessed before, during, and after exposure by asking participants to describe their present arousal and valence with the help of the SAM figures that were hung on the mirror.

### Data analysis

The analyses were run using the IBM Statistical Package for the Social Sciences (SPSS, version 28.0). Plausibility checks were performed for all variables. For this purpose, box plots created in SPSS were inspected for signs of obvious errors in *f*0 extraction. There were no extreme outliers (>three times the interquartile range). Moreover, Mahalanobis distance analysis only identified one participant’s vocal data as an outlier, but since this was due to a naturally high *f*0 we retained this participant’s responses in the dataset. Regarding the final dataset, sample characteristics were analyzed descriptively. Assumptions for the *t*-test for dependent variables were met and the robustness of bivariate normal distribution for correlations was presumed [cf. (75)]. The hypothesized increase in *f*0 during body exposure compared to baseline was likewise tested using a (two-tailed) *t*-test for dependent variables, separately for *f*0_mean_ and *f*0_variabilitymeasures_. Furthermore, to test the influence of trait-like eating disorder severity on *f*0, we conducted linear regression analyses separately for *f*0_mean_ and *f*0_variabilitymeasures_ as dependent variable and trait-like questionnaires as independent variables. Regarding state measures, Pearson’s product-moment correlations were used to examine the relationship of *f*0 with self-reported state body-related distress (BISS), arousal and valence (SAM), and positive and negative affect (PANAS-X). To compare self-reported data with *f*0, for the SAM, we averaged arousal and valence measures using data from before, during, and after each condition; for the BISS and the PANAS-X, we aggregated data from before and after each condition. Pearson’s product-moment correlations between *f*0 and HR were additionally calculated. As a manipulation check for HR, to ensure that the task indeed elicited an HR response, we tested the difference between baseline and body exposure for HR_mean_ and HR_maximum_ using a (two-tailed) *t*-test for dependent variables. Effect sizes were classified as small (|*d*|= 0.2), moderate (|*d*|= 0.5), and large (|*d*|= 0.8) in line with Cohen [(76), pp.77–83]. For all analyses, the significance level was set at α = 0.05, with Bonferroni-Holm alpha-level corrections (77) applied to account for multiple testing.

## Results

### Participant characteristics

Participants’ characteristics revealed a mean age in the early twenties (*M*: 23.1, *SD*: 3.2; range: 18–36), a mean BMI in the normal-weight range (*M*: 21.3 kg/m^2^, *SD*: 2.8 kg/m^2^; range: 18.2–37.9 kg/m^2^), and an average amount of exercise per week (*M*: 4.4 h, *SD*: 2.3 h; range: 1–8 h) compared to the general population (78). Likewise, the trait-like eating disorder severity and state body dissatisfaction (presented in Table 2) lay within the usual range for women without eating disorders (59, 64). Participants’ physiological characteristics are depicted in Table 3. For voice, the *f*0 ranged from 103.1 to 284.2 Hz in the baseline condition and from 110.2 to 285.81 Hz in the body exposure condition, which lies within the range of female *f*0 (70). HR ranged from 56 to 116 bpm during the baseline condition and from 65 to 123 bpm during the body exposure condition, indicating normotonic values within the sample [(79), (p. 12)].

**TABLE 2:** Means and standard deviations of study-relevant trait-like and state measures.

Variable	Subscale	Preliminary			Condition					
					**Baseline**			**Body exposure**		
Trait-like measures		*M*	*SD*	*Range*						
**EDE-Q^a^**										
	Global score	0.9	0.7	0.3–3.1						
	Shape concern	1.2	0.8	0.2–4.0						
	Weight concern	0.9	0.8	0.2–3.1						
	Restraint	1.0	1.0	0.1–3.6						
	Eating concern	0.5	0.6	0.1–2.6						
**EDI-2^b^**										
	Drive for thinness	2.7	0.7	1.7–4.9						
	Body dissatisfaction	3.4	0.3	1.4–4.2						
State measures					*M*	*SD*	*Range*	*M*	*SD*	*Range*
**SAM^c,d^**										
	Arousal				3.0	1.2	1.0–6.0	3.9	1.6	1.0–7.4
	Valence				5.9	1.3	3.0–8.0	6.0	1.3	2.4–9.0
PANAS^e,f^										
	Positive affect				2.5	0.6	3.1–4.5	2.4	0.8	3.2–4.3
	Negative affect				1.4	0.4	2.3–3.3	1.4	0.4	2.2 – 3.4
BISS^f,g^					6.4	1.4	5.7–8.1	5.9	1.3	6.0–8.0

^a^Eating Disorder Examination-Questionnaire.

^b^Eating Disorder Inventory-2.

^c^Self-Assessment Manikin.

^d^Average of assessments before, during, and after each condition.

^e^Positive and Negative Affect Schedule.

^f^Average of assessments before and after each condition.

^g^Body Image States Scale.

**TABLE 3:** Means and variabilities (standard deviations) of psychophysiological measures.

Variable	Index	Baseline	Body exposure
Fundamental frequency^a^	*M (SD)*	204.7 (20.5)	209.1 (19.8)
	*Variability (SD)*	71.9 (25.4)	73.0 (24.6)
Heart rate^b^	*M (SD)*	84.5 (10.8)	86.7 (11.0)
	*Maximum (SD)*	99.4 (10.3)	107.5 (11.0)

^a^In Hz.

^b^In beats per minute.

### Increase of fundamental frequency during body exposure compared to baseline

With respect to the first hypothesis, paired-samples *t*-tests revealed a significant increase from baseline to body exposure for *f*0_mean_, indicating higher vocally encoded arousal during exposure sessions for *f*0_mean_ [*t*(72) = –3.96, *p* ≤ 0.001, *d* = 0.46]. However, for *f*0_variabilitymeasures_, we did not find statistically significant differences after Bonferroni-Holm correction [for *f*0_dispersion_ *t*(72) = –0.39, *p* = 0.694, *d* = 0.05; for *f*0_range_ *t*(72) = –0.77, *p* = 0.223, *d* = 0.09_;_ for *f*0_SD_ *t*(72) = –1.09, *p* = 0.140, *d* = –0.13].

### Prediction of fundamental frequency by trait-like eating disorder severity

Regarding the severity of eating disorder symptoms as a predictor of Δ*f*0_mean_, the multiple correlation of *R* = 0.39 was found to be statistically significant [*F*(3,68) = 4.17, *p* = 0.009]. Furthermore, a higher EDE-Q_global_ score led to higher *f*0 (β = 6.22, *p* = 0.026), while no significant predictions emerged for the EDI-2 subscales Body Dissatisfaction (β = 6.11, *p* = 0.116) and Drive for Thinness (β = –1.30, *p* = 0.623). Regarding the severity of eating disorder symptoms as a predictor of Δ*f*0_variabilitymeasures_, no significant results emerged. Thus, for Δ*f*0_dispersion_, the multiple correlation of *R* = 0.21 was not found to be statistically significant [*F*(3,68) = 1.08, *p* = 0.361]. Moreover, the EDE-Q_global_ (β = –3.06, *p* = 0.681) and the EDI-2 subscales Body Dissatisfaction (β = –10.36, *p* = 0.320) and Drive for Thinness (β = –4.06, *p* = 0.570) did not contribute significantly to the prediction of Δ*f*0_dispersion_. Likewise, regarding Δ*f*0_range,_ the multiple correlation of *R* = 0.28 was not found to be significant [*F*(6,65) = 2.84, *p* = 0.464] as neither were the EDE-Q_global_ (β = –7.80, *p* = 0.420) as well as the EDI-2 subscales Body Dissatisfaction (β = –2.42, *p* = 0.856) and Drive for Thinness (β = –2.89, *p* = 0.757). Also, regarding Δ*f*0_SD,_ the multiple correlation of *R* = 0.27 was not statistically significant [*F*(6,65) = 2.73, *p* = 0.519]. Thus, no significant predictions could be done for the EDE-Q_global_ (β = 3.14, *p* = 0.667) or the EDI-2 subscales Body Dissatisfaction (β = 15.78, *p* = 0.121) and Drive for Thinness (β = –3.16, *p* = 0.964).

### Correlations between physiological variables and state questionnaire data

In terms of convergent validity, state body-related distress (BISS) was not significantly correlated with Δ*f*0_mean_ (*r* = 0.14, *p* = 0.218) or with Δ*f*0_variabilitymeasures_ (for Δ*f*0_dispersion_: *r* = –0.17, *p* = 0.141; for Δ*f*0_range_: *r* = –0.32, *p* = 0.792; for Δ*f*0_SD_: *r* = –0.13, *p* = 0.264). Regarding the correlations of *f*0 and questionnaire-based arousal (SAM) averaged over the course of body exposure (i.e., before, during, and after body exposure), Δ*f*0_mean_ yielded significant positive correlations (*r* = 0.30, *p* = 0.026), while no significant correlation was shown between arousal and Δ*f*0_variability_ _measures_(for Δ*f*0_dispersion_: *r* = –0.22, *p* = 0.058; for Δ*f*0_range_: *r* = –0.07, *p* = 0.554; for Δ*f*0_SD_: *r* = –0.01, *p* = 0.944). Regarding questionnaire-based valence (SAM) averaged across the three time stamps, Δ*f*0_mean_ correlated significantly negatively with valence (*r* = –0.34, *p* = 0.009), but again, no significant correlations were found for Δ*f*0_variabilitymeasures_ (for Δ*f*0_dispersion_: *r* = 0.11, *p* = 0.353; for Δ*f*0_range_: *r* = 0.57, *p* = 0.629; for Δ*f*0_SD_: *r* = 0.15, *p* = 0.193). Regarding affect, no statistically significant results emerged when applying Bonferroni-Holm corrections. Thus, neither Δ*f*0_mean_ (*r* = 0.15, *p* = 0.192) nor Δ*f*0_variabilitymeasures_ (Δ*f*0_dispersion_: *r* = 0.11, *p* = 0.361; Δ*f*0_range_: *r* = –0.01, *p* = 0.992; Δ*f*0_SD_: *r* = 0.21, *p* = 0.082) correlated significantly with General Positive Affect (PANAS-X). Likewise, neither *f*0_mean_ (*r* = 0.23, *p* = 0.050) nor Δ*f*0_variabilitymeasures_ (Δ*f*0_dispersion_: *r* = –0.33, *p* = 0.075; Δ*f*0_range_: *r* = –0.39, *p* = 0.073; Δ*f*0_SD_: *r* = –0.12, *p* = 0.299) correlated significantly with General Negative Affect (PANAS-X).

Further, in terms of the relationship of *f*0 with HR, no significant results emerged. Regarding ΔHR_mean_, neither Δ*f*0_mean_ (*r* = 0.15 *p* = 0.207) nor Δ*f*0_variabilitymeasures_ (Δ*f*0_dispersion_: *r* = 0.03 *p* = 0.796; Δ*f*0_range_: *r* = 0.06, *p* = 0.620; Δ*f*0_SD_: *r* = –0.06, *p* = 0.614) correlated significantly with ΔHR_mean_. Also, regarding ΔHR_maximum_, no significant correlations were found for Δ*f*0_mean_ (*r* = 0.06 *p* = 0.602) or Δ*f*0_variabilitymeasures_ (Δ*f*0_dispersion_: *r* = 0.08 *p* = 0.506; Δ*f*0_range_: *r* = 0.16, *p* = 0.167; Δ*f*0_SD_: *r* = –0.13, *p* = 0.284). However, as with Δ*f*0_mean_, HR increased during body exposure [for HR_maximum_: *t*(71) = –2.09, *p* = 0.040, *d* = 0.25; for HR_mean_: *t*(71) = –5.80, *p* ≤ 0.001, *d* = 0.69] compared to baseline.

## Discussion

The aim of the present study was to validate *f*0 _mean_ and *f*0_variabilitymeasures_ as correlates of vocally encoded arousal, valence, and body-related distress. To achieve this, healthy women underwent a 3-min neutral, non-body-related baseline condition and a subsequent 7-min body exposure session depicting an experimentally induced stressor. Both indices of *f*0 _mean_ and *f*0_variabilitymeasures_ have been used previously in different stress-provoking tasks but the results have been inconclusive overall. While higher self-reported arousal led to higher *f*0_mean/variability_ in some studies [e.g., (38, 41)], it led to lower *f*0_mean/variability measures_ in others [e.g., (30, 34)]. In line with our first hypothesis, for *f*0_mean_, we found the predicted increase during body exposure compared to baseline, providing a first indication that *f*0_mean_ is influenced by psychological distress. However, regarding *f*0_variabilitymeasures_, we did not find the expected decrease or any differences between the baseline and body exposure condition, indicating that the induced stressor of body exposure was not evident in *f*0_variabilitymeasures_.

In terms of our second hypothesis, only one of two measures of trait-like eating disorder severity was found to be significant, with higher trait-like severity emerging as a predictor of higher *f*0_mean_. Again, no associations were found for *f*0_variabilitymeasures_. Therefore, it cannot be conclusively stated that *f*0 is a parameter of trait-like eating disorder severity. These findings corroborate the results of previous research: In a study in persons with binge eating disorder, lower *f*0_mean_ was associated with higher trait-like body dissatisfaction (34), whereas in line with our study on eating disorder severity, a study in a sample of females with overweight and obesity reported that higher *f*0_mean_ correlated with higher trait-like body dissatisfaction (35). This demonstrates the unclear direction of *f*0 as a correlate of trait-like eating disorder severity, which is potentially related to the different samples of clinical persons [i.e., females with binge eating disorder; (34)] and samples of individuals without mental disorders [i.e., females without mental disorders in our study, females with overweight/obesity; (35)].

Third, regarding the state parameters, the expected associations of *f*0_mean/variability measures_ with state body dissatisfaction were not found in the present study. This is in line with the lack of correlation between *f*0_mean_ and state body dissatisfaction in females with binge eating disorder reported by Baur and colleagues (34), but is in contrast to the negative correlation between state dissatisfaction and *f*0_mean_ in females with overweight found in another study by Baur and colleauges (35). As a whole, no clear pattern emerges regarding *f*0 in terms of trait-like and state body-related distress. Therefore, *f*0 may not be suitable as a marker of distinct clinical constructs such as body-related distress, social anxiety disorder (54), or pathological fear (30), but may potentially be viewed as a broader correlate of arousal and valence.

Following the pattern of findings reported for the first hypothesis, the expected positive association between *f*0_mean/variability measures_ and self-defined arousal and the negative association with valence were only evident for *f*0_mean_ and not for *f*0_variability mesasures_. Regarding arousal, our results – in line with previous literature on anxiety exposure (54) and body exposure (35) – provide further indication that *f*0_mean_ is a correlate of vocally encoded arousal. With regard to valence, the correlation with *f*0_mean_ yields more evidence that not only specific emotional states [e.g., fear, (29)], but also general valence, should be considered as correlates of *f*0_mean_. Therefore, future studies should analyze affect in *f*0 on the bipolar dimension of arousal and valence (33). Contrary to our assumption, there were no significant correlations between general affect and *f*0_mean/variability measures_. In part, this contrasts with our findings on arousal and valence, which are both commonly seen as dimensions of affect (80). Hence, based on our inconclusive results, *f*0 cannot be clearly seen as a correlate of general affect. Fourth, the findings did not reveal the hypothesized positive association between *f*0_mean/variability measures_ and HR_mean/maximum_, although greater activation was shown in both psychophysiological parameters in the body exposure condition compared to baseline.

The finding that both *f*0 and HR increased from baseline to body exposure is in line with several experiments on stress-inducing tasks [e.g., for *f*0: (38), for HR: (81)]. However, the lack of correlation between the two psychophysiological measures is unexpected, as theoretically, changes in *f*0 should (among other factors) be caused by cyclic changes in heartbeat (82). Further, positive associations between *f*0 and HR were found during other laboratory stressors [i.e., arithmetic mental stress task (83); or during a couple’s conflict about a problematic relationship topic (22)]. This is in contrast to the non-significant correlations of *f*0 and HR during body exposure found in the present study. One possible explanation for this finding may be that both markers seem to be dependent on the distinct stressor that is used to provoke arousal. For instance, Alvear and colleagues (83) found a stronger association between *f*0 and HR under stress induced by cognitive load (i.e., subtracting units from a number) compared to stress induced by physical stressors (i.e., cold pressor test). Moreover, the distinct variable assessed may influence the association, as *f*0 was unrelated to systolic, diastolic and mean blood pressure, but was related to HR (83). In addition, it has not yet been resolved which precise physiological mechanisms are responsible for the association between the two measures (83). To further explore the relationship between these two psychophysiological parameters, future investigations should include different stressors and different cardiovascular measures.

Further, the null findings for *f*0_varability measures_ on all variables of our study indicate that the interpretation on *f*0_varability measures_ lacks a clear direction. From a theoretical perspective, body-related distress is assumed to create sympathetic arousal, leading to a decrease in *f*0_varability measures_ in stressful situations (18) such as body exposure. Moreover, our results are thus in contrast to Hagenaars and van Minnen (30), who reported negative associations between *f*0_varability measures_ and the specific emotional state of fear in patients with panic disorder with agoraphobia. Nevertheless, the lack of effects regarding *f*0_varability measures_ in the present study underline the inconsistent results in the literature [e.g., (4)], with some studies reporting increased *f*0_varability measures_ in response to arousal and valence created using laboratory stressors (28), others reporting decreased *f*0_varability measures_ (30), and some finding no correlation in this regard (84).

The present study was the first to examine arousal, valence, and general affect as depicted by *f*0_mean_ and *f*0_varability measures_ during body exposure. Some limitations need to be taken into consideration when interpreting the results: First, methodically, from our correlational findings, we are unable to draw conclusions regarding causality in the sense of a causal link between the psychophysiological cues of *f*0 and HR and the experience of arousal in the body exposure task. Moreover, the study might have lacked statistical power, because the sample was relatively small and the results showed mostly small to medium effects. With a larger sample size, effects might have become more visible or additional effects might have been detected. In addition, future studies should address the potential relationship between body size (i.e., height and weight) and *f*0. However, the literature is inconclusive: A recent metanalysis on 39 independent samples referring to this topic found that the relationship between *f*0 and height/weight accounted for only less than 2% variance within individuals (85). We retrospectively calculated the correlation between *f*0_(all indices)_ and BMI and also found no significant effects in our study.

Furthermore, although we implemented a neutral baseline condition, we did not counterbalance the two conditions of baseline and body exposure, and therefore cannot rule out an order bias. However, body exposure is well researched as a suitable stressor, with previous studies demonstrating heightened levels of self-reported arousal (48) and body-related distress (49) in response to body exposure. Second, we examined emotions only in terms of general affect, arousal, and valence rather than analyzing specific emotions, whereas some studies reported a different pattern of *f*0 with regard to individual emotions such as anger [e.g., (19)], sadness [cf. (30)], or disgust [e.g., (86)]. We chose to stick to the two dimensions of arousal and valence because this bipolar scale has been used to measure affect in other questionnaire-based studies [e.g., (65)]. Moreover, the investigation of individual emotions may yield ambiguous findings due to the difficulty of differentiating between distinct emotions such as anger and sadness from one particular *f*0 pattern [cf. (18)]. Third, our sample only comprised Caucasian women without mental disorders, and future studies should therefore consider a more heterogeneous and potentially clinical sample. However, research on vocally encoded affect found comparable results across gender (87) and different ethnic groups (88), indicating that our findings might be transferred to different samples. Furthermore, some studies hint at an influence of phonological differences with regard to the language being spoken [(89); *f*0_range_], while others indicate that differences in *f*0 between languages might rather be a cultural artifact [(90); *f*0_mean_]. To contribute to resolving this debate, our study should be replicated in samples with other languages. Fourth, future research should consider validating *f*0 based on further acoustic parameters such as speech rate (30), amplitude (91), or formant frequencies (F_1_, F_2_; quality of voice; (92), and further on additional psychophysiological measures such as eye-tracking [e.g., 93), cortisol (24), blood pressure (42)], or neurological aspects [e.g., neural network-based approaches, (94)]. Finally, we utilized a wrist monitor as a non-invasive measure of HR. Although the device has shown appropriate validity and reliability in other studies (95), other specific instruments to assess HR or HR variability [e.g., electrocardiogram, (52); automatic cuffs for blood pressure, (42)] should be considered, albeit with the potential cost of distracting participants from the assigned task.

In summary, the present study contributes to research on vocal analyses of affect, as only *f*0_mean_, but not *f*0_varability measures_, emerged as a valid marker of vocally encoded arousal and valence. We further suggest that *f*0_mean_ represents a valid global marker of emotional arousal and valence rather than of concrete body-related distress. Due to its economical and non-invasive nature (17, 96, 97) and – as our study shows – sufficient validity, the analysis of *f*0_mean_ might be used prospectively as an adjunctive psychophysiological measure to examine affect in a manner that is less biased than conventional methods (16).

## Data availability statement

The raw data supporting the conclusions of this article will be made available by the authors, without undue reservation.

## Ethics statement

This study involving human participants was reviewed and approved by the Ethics Committee of Osnabrück University (4/71043.5). The patients/participants provided their written informed consent to participate in this study.

## Author contributions

VO: data collection, analysis, writing—original draft, and review and editing. JT and SV: conceptualization and design. JB and JS: data–vocal analysis. AH: conceptualization. All authors contributed to the revision of the manuscript, read, and approved the submitted version.
